# Prospects of Phage Application in the Treatment of Acne Caused by *Propionibacterium acnes*

**DOI:** 10.3389/fmicb.2017.00164

**Published:** 2017-02-08

**Authors:** Ewa Jończyk-Matysiak, Beata Weber-Dąbrowska, Maciej Żaczek, Ryszard Międzybrodzki, Sławomir Letkiewicz, Marzanna Łusiak-Szelchowska, Andrzej Górski

**Affiliations:** ^1^Bacteriophage Laboratory, Ludwik Hirszfeld Institute of Immunology and Experimental Therapy, Polish Academy of SciencesWroclaw, Poland; ^2^Phage Therapy Unit, Ludwik Hirszfeld Institute of Immunology and Experimental Therapy, Polish Academy of SciencesWroclaw, Poland; ^3^Department of Clinical Immunology, Transplantation Institute, Medical University of WarsawWarsaw, Poland; ^4^Medical Sciences Institute, Katowice School of EconomicsKatowice, Poland

**Keywords:** *Propionibacterium acnes* phages, experimental phage therapy, treatment of infections, antibiotic resistance, topical application

## Abstract

*Propionibacterium acnes* is associated with purulent skin infections, and it poses a global problem for both patients and doctors. Acne vulgaris (acne) remains a problem due to its chronic character and difficulty of treatment, as well as its large impact on patients' quality of life. Due to the chronic course of the disease, treatment is long lasting, and often ineffective. Currently there are data regarding isolation of *P. acnes* phages, and there have been numerous studies on phage killing of *P. acnes*, but no data are available on phage application specifically in acne treatment. In this review, we have summarized the current knowledge on the phages active against *P. acnes* described so far and their potential application in the treatment of acne associated with *P. acnes*. The treatment of acne with phages may be important in order to reduce the overuse of antibiotics, which are currently the main acne treatment. However, more detailed studies are first needed to understand phage functioning in the skin microbiome and the possibility to use phages to combat *P. acnes*.

## Introduction

We are entering the post-antibiotic era: infections formerly easy to cure are becoming difficult to treat, as both increasing frequency of treatment failure and increasing severity of infections have been observed (Jassim and Limoges, 2014; WHO, 2014). The number of new approved antibiotics has dramatically decreased, and research on these new drugs is difficult, arduous, and unprofitable (Clarke, 2003; WHO, 2014).

At present, ~50 million people in the US are suffering from acne, 85% of whom are at the age of 12–25 (Sidbury and Paller, 2000; Lynn et al., 2016). Disorders arising as a result of the reaction to acne can lead to a significant reduction in self-esteem (Sidbury and Paller, 2000). The inflammatory form of acne may leave scars that can result in permanent disfigurement. Correct and appropriate use of antibiotics in the treatment of acne will help to preserve their utility in the face of increasing antibiotic resistance, and therefore greater awareness of the issues is required among prescribing physicians (Dréno, 2016). The chief problem with antibiotic therapy of acne is the common tendency to prolonged use and overuse of antibiotics. Therefore, it is necessary to treat acne with effective alternatives to commonly used antibiotics to reduce the likelihood of resistance to this type of treatment (Walsh et al., 2016) and obtain a highly specific agent to destroy bacteria effectively. The US National Institutes of Health (NIH, US) indicates phages to be innovative components that may be used to combat antibiotic resistance (NIH, 2014).

Detailed understanding and expanded knowledge of the microbiome, especially that of human skin, may be fundamental for recognizing skin-associated pathogenesis, especially acne vulgaris, and finding therapeutic solutions (Marinelli et al., 2012), e.g., the possibility to develop phage therapy of this disease. Further and more detailed studies are needed to understand phages' function in the skin microbiome and their participation in resistance gene transfer (Hannigan and Grice, 2013).

## Pathogenesis and epidemiology of acne

Acne vulgaris is the most common human skin disease (Valente Duarte de Sousa, 2014). Despite the implementation of new-generation antibiotics, the disease continues to have a social dimension. According to previous studies, the skin of teenagers suffering from acne vulgaris has up to 100-fold higher numbers of *Propionibacterium acnes* compared to healthy skin (Leyden et al., 1975). However, subsequent studies have failed to detect any significant discrepancies in the number of *P. acnes* between patients and controls. The observed levels of *P. acnes* were almost identical, but increased levels of *S. epidermidis* were observed (Bek-Thomsen et al., 2008). The presence of specific phylotypes of *P. acnes* is thought to be associated with the disease rather than the abundance of *P. acnes* (Tomida et al., 2013).

The etiology of acne is multifactorial, but probably the main agent associated with acne is the bacterium *P. acnes*. This pathogen is associated with acne, but the causality of the disease is not clear, and the exact role of *P. acnes* in acne is controversial. Acne is a chronic dermatosis that may cause lesions observed as papules or nodules (Zaenglein et al., 2016). The clinical form of acne depends on the interaction of the following factors: malfunctioning shedding of hair follicle cells, excessive sebum secretion, colonization of hair follicles by *P. acnes*, and individual factors depending on the host (e.g., the status of the immune system; Sidbury and Paller, 2000). Oversecreted sebum is accumulated under interconnected keratinocytes which clog the outlet of the sebaceous glands, thus creating ideal conditions for *P. acnes* growth. These bacteria trigger inflammatory infiltration, and the inflammation can spread to the dermis. A severe form of acne is acne inversa (also known as hidradenitis suppurativa), a chronic inflammatory disease which has the most significant impact on patients' quality of life among all assessed dermatological diseases (Wollina et al., 2013). It may be observed especially in patients with altered immunity with deficiency of antimicrobial protein secretion (Wolk et al., 2011). Pain is a major symptom of this form of acne. Treatment is by application of a drug, sometimes even combined with surgery (Wollina et al., 2013).

*Propionibacterium* is one of the main components of human skin microbiota of healthy adults (Human Microbiome Project Consortium, 2012; Hannigan and Grice, 2013). It predominates in sebaceous regions and is estimated to represent nearly 90% of the microbiota (Fitz-Gibbon et al., 2013). It is an opportunistic, Gram-positive, microaerophilic, nonmotile, and fat splitting bacteria, which—due to its tendency to elicit an inflammatory response—is thought to be the probable main cause of acne. The genomes of different *P. acnes* strains were found to be of similar sizes, G+C contents, and encode a similar number of open reading frames (ORFs) (Tomida et al., 2013). Another sequencing analysis suggested that acquired DNA sequences of *P. acnes* and its immune elements were important in determining the virulence of *P. acnes*, and these elements should be the therapeutic target (Fitz-Gibbon et al., 2013). According to Fitz-Gibbon et al. (2013), genomic comparison of *P. acnes* strains indicated that there may be specific genes that contribute to the pathology of acne. Holland et al. (2010) reported that *P. acnes* produces hydrolases likely to be involved in degrading human tissue components and immunoreactive adhesins which are expected to play a role in its virulence. Moreover, factors that have only been suggested to play a part in *P. acnes* pathogenesis (there is no experimental evidence) are: co-hemolytic CAMP factor 5, gehA lipase, putative hemolysin tly, sialidases, neuraminidases, endoglycoceramidases (Brüggemann, 2005). The facts that *P. acnes* may persist inside macrophages (prostate-infiltrating macrophages and the human macrophage cell line THP-1) and that it survives phagocytosis are very important for the pathogenesis of other diseases associated with this pathogen. This phenomenon may be important for *P. acnes*-associated inflammatory diseases (Fischer et al., 2013).

## Conventional treatment of acne

Antibiotics (both oral and topical) have been used as therapeutics for acne treatment for 40 years. It is estimated that every year ~5 million prescriptions of antibiotics for oral treatment of acne are prescribed (Stern, 2000). In recent years, topical, enteral, and parenteral antibiotics have been used. The most commonly used are: nadifloxacin, ofloxacin, erythromycin, clindamycin hydrochloride, doxycycline, tetracycline hydrochloride, minocycline, ampicillin, cephalexin, gentamycin, and trimethoprim-sulfamethoxazole (Nishijima et al., 1996; Michałek et al., 2015). Currently, experimental trials are underway on a new generation quinolone—ozenoxacin *in vitro* (Choudhury et al., 2011). This quinolone is mainly for topical administration which is used as effective treatment for complicated skin and soft-tissue infections. Its mechanism is based on simultaneous affinity for two enzymes: DNA gyrase and topoisomerase IV (Karpiuk and Tyski, 2013). Due to the chronic course of acne vulgaris, treatment is long and often ineffective. Ozenoxacin application leads to shortened time of treatment. For the same reason, oral antibiotic therapy is supported by drugs applied topically, e.g., doxycycline (100 mg), which is administered orally once daily for 12 weeks and combined additionally with 5% dapsone applied topically twice a day (Kircik, 2016). Nagler et al. (2016) reported that the average duration of antibiotic therapy in acne in some cases may even exceed 1 year (Nagler et al., 2016). Such prolonged therapy can be shortened by the addition of isotretinoin (a 13-cis-retinoic acid which is a non-aromatic retinoid; Tilles, 2014), whose action is associated with reduction of sebum production, anti-inflammatory properties, reduction in *P. acnes* and an effect on comedogenesis (decreasing hyperkeratinization; Layton, 2009). Isotretinoin is used to treat severe and recalcitrant cases of inflammatory acne. This drug significantly improves the results of acne treatment, but its application is associated with such adverse side effects as the ability to cause mental disorders, e.g., depression or suicidal thoughts, and even incidents of suicide have been reported. Nevertheless, improvement in mood and an increase in the quality of patients' lives after its application have been observed (Schrom et al., 2016).

Since prolonged antibiotic therapy in acne treatment is common, antibiotic resistance of *P. acnes* strains has been extensively observed (Sardana et al., 2016; Walsh et al., 2016). In 1976 there were no reports of antibiotic resistance in *Propionibacterium* strains (Leyden, 1976). But already 3 years later bacterial strains isolated from skin of patients with acne proved to be cross-resistant to erythromycin and clindamycin in both *in vitro* and *in vivo* studies (Crawford et al., 1979). Scientists and physicians have raised the alarm that an alternative to topical antibiotics should be found (Dréno, 2016). They suggest limiting the use of antibiotics, and especially avoiding their use as monotherapy (Zaenglein et al., 2016). It has been demonstrated that prolonged use of antibiotics generates selective pressure to induce antibiotic-resistant bacteria, and it correlates with the duration of these drugs' usage. Antibiotic resistance in these bacteria develops by spontaneous mutation rather than the horizontal transfer of multiple drug resistance genetic determinants (Moore and Sauer, 2008; Neely et al., 2008).

Using antibiotics may be associated with disturbance in natural microbiota, and it may cause the risk of colonization of such stains as *Streptococcus pyogenes* (Levy et al., 2003). Another danger that may result from application of antibiotics is the possibility of *P. acnes* to form an antibiotic-resistant biofilm. This natural state of bacterial colonization may protect bacteria against antimicrobials and facilitate bacterial adherence to tissue (Coenye et al., 2008; Portillo et al., 2013; Dréno, 2016). *P. acnes* has been shown visually to exist as biofilms on the skin by Jahns and Alexeyev (2014), who observed biofilm spreading for 1900 μm in a terminal hair follicle. To obtain the optimal absorption of e.g., tetracycline, it is recommended to be used on an empty stomach (Sidbury and Paller, 2000). It may cause an increase in the risk of occurrence of such adverse effects as gastrointestinal disorders. Moreover, in the case of women, candidiasis may develop. Also, acne vulgaris is thought to have an influence on the incidence of suicidal ideation in acne patients (~7.1%; Kumar et al., 2016).

For the topical treatment of acne, antibiotic-containing creams, gels, solutions, and microemulsions are used (Sidbury and Paller, 2000).

## Bacteriophages

Bacteriophages are bacterial viruses that naturally control microbial populations. They can multiply only in bacterial cells, and therefore may be active at the site of infection, where pathogenic bacteria are present. It is estimated that in the biosphere bacteriophages are 10 times more frequent than bacterial cells (Abedon, 2011). They are commonly found in the biosphere (Lin et al., 2010; Zhan et al., 2015) and human and animal organisms (Keller and Traub, 1974; Caroli et al., 1980; Gantzer et al., 2002; Bachrach et al., 2003; Reyes et al., 2012). Bacteriophage morphology shows great diversity, and their classification was traditionally based on the shape of the phage particle and the type of nucleic acid. However, the similarity in structure does not determine the biological properties of bacteriophages. Nowadays the phage classification is mainly based on DNA sequence identity (Kropiński et al., 2016).

Phages are called “living drugs” (Jassim and Limoges, 2014), and this term reflects the behavior of phages at the infection site. There are data confirming their efficacy in treating local and systemic infections caused by antibiotic-resistant bacterial strains (including those where bacteria resistant to multiple antibiotics are etiological agents; Biswas et al., 2002; Keen, 2012; Międzybrodzki et al., 2012; Chhibber et al., 2013; Borysowski et al., 2014; Rose et al., 2014; Cao et al., 2015; Sarker et al., 2016).

Phages have features that give them advantages over antibiotics, e.g., they are specific to their bacterial host (Ly-Chatain, 2014), which may minimize the probability of appearance of secondary infection (Golkar et al., 2014), and they multiply at the site of infection where there are sensitive bacteria (Loc-Carrillo and Abedon, 2011). The development of resistance of bacteria to antibiotics does not equate to simultaneous development of phage resistance in bacteria, although bacterial resistance to phages may also be observed. However, phage-resistant mutants that had lost phage receptors on the cell surface proved to be less pathogenic than phage-susceptible ones (Capparelli et al., 2010; León and Bastías, 2015). Phages are commonly found in the biosphere and therefore are environmentally friendly, because they are natural structures based on natural selection (Golkar et al., 2014). The isolation of new phages for therapeutic purposes is an affordable and rapid process compared to research and development of new antibiotics (which takes several years and may cost millions of dollars for clinical trials). Moreover, phage therapy is less expensive than conventional antibiotic therapy, which has been demonstrated in the case of patients with staphylococcal infections (Międzybrodzki et al., 2007).

Phages are proved to be safe and well tolerated, with few side-effects and without any toxic effects (Bruttin and Brüssow, 2005; Borysowski and Górski, 2008; Denou et al., 2009; Międzybrodzki et al., 2012; Miernikiewicz et al., 2013; Łusiak-Szelachowska et al., 2014).

An important aspect from the therapeutic point of view is the ability of phages to be temperate (e.g., co-exist with their host, either in a lysogenic, or pseudolysogenic state), as well as their ability to induce transduction. These processes may result in gene transfer between bacterial strains, as well as antibiotic resistance genes or pathogenicity genes. These factors are critical for the safety of phage therapy. However, Modi et al. (2013) based on their research on antibiotic treatment of mice (ciprofloxacin or ampicillin) suggested that antibiotic application increased the frequency of phage integration into the bacterial genome, and enriched the phage metagenome for stress and niche specific functions, which shaped the phage-bacterial network to potentiate accessibility of phage genetic elements. Moreoever, the phage metagenome from mice treated with antibiotics was enriched with additional functions that may contribute to metabolism of the host (e.g., ampicillin-treated mice acquired a broader carbohydrate metabolic pathway).

To take advantage of the maximal phage potential, a regulatory framework should be established (Jassim and Limoges, 2014). Successful phage therapy requires phages of only proven lytic activity, whose lifecycle results from the burst size (Mirzaei and Nilsson, 2015). As mentioned above, especially personalized phage therapy (tailored therapy) that uses phages adjusted to specific pathogenic bacteria that are the cause of infection may be successful (Jassim and Limoges, 2014; Mattila et al., 2015) due to higher (5–6-fold) success rates (Zhukov-Verezhnikov et al., 1978) compared to *prêt-à-porter* preparations. It is proposed to develop improved methods of administration and new formulations that reduce the exposure of phage to destructive conditions. As emphasized by Nilsson (2014), phages intended to be used in phage therapy need to be well characterized, produced under appropriate conditions to obtain the required titer, validated, purified, and approved for clinical use. This will create a chance to minimize the problem of bacterial resistance to antibiotics. Thus, it is necessary to conduct phase III clinical trials (Vandenheuvel et al., 2015).

Table 1 summarizes phage topical application in the therapy of skin infections. All the described studies (Table 1) concern infections caused by bacteria other than *P. acnes*. These findings show that topical phage therapy in both animals and humans may be effective, so there are strong indications supporting the potential of *P. acnes* phages to also treat acne.

**Table 1:** **Topical phage therapy of infection**.

**Pathogen**	**Model**	**Outcome**	**Result of the therapy**
*Staphylococcus aureus*	Early studies of the application of phage therapy in dermatology in 143 patients with purulent skin infections.	Phage application as direct injection into the wound and surrounding tissue.	The best results were observed in patients with acute infections of deep skin. In the studied group of patients successful treatment was observed in 75%, improvement in 7.7%, and no effect of the therapy was observed only in 4.9% of the treated patients (Beridze, 1938).
*Staphylococcus*	55 patients with furunculosis.	Oral and local phage administration.	In all cases good therapeutic results were obtained (Ślopek et al., 1987).
*Staphylococcus, Pseudomonas, Klebsiella, Proteus, Escherichia*	Studies concerned 31 patients with suppurative skin infections.	The treatment lasted 2–16 weeks.	During the treatment an improvement with suppression of local inflammation, faster healing of ulcers, and eradication of bacteria was observed. Good therapeutic effects were obtained in the case of 25 patients (16 with outstanding results, 7 with marked improvement, and 2 with transient improvement; Cisło et al., 1987).
*Klebsiella pneumoniae* B5055	Mouse model of burn wound infection.	A single dose of topical application of the Kpn5 phage suspended in 3% hydrogel (at MOI of 200) used as ointment.	Mice treated with only a single dose of phage showed a significant reduction in animals' mortality (66%) compared to the control group (Kumari et al., 2010).
*Staphylococcus aureus, Pseudomonas aeruginosa, Acinetobacter baumannii*.	Animal models of diabetic cutaneous wound infection.	Topical administration in combination with wound debridement	A decrease in bacterial counts and improved wound healing in a rodent model of *Staphylococcus aureus* and *Pseudomonas aeruginosa* infections. The therapy was not as effective against *Acinetobacter baumannii*. Bacteriophage treatment may be effective in resolving chronic infections also when applied in combination with wound debridement (Mendes et al., 2013).
*Staphylococcus, Streptococcus, P. aeruginosa*	Patients with wounds/ulcers.	Local administration of PhagoBioDerm, which contains ciprofloxacin, α-chymotrypsin benzocaine, and bacteriophage based on biodegradable poly(ester amide)s matrix.	Resulted in healing in the case of 70% of patients. It was associated with elimination of and/or reduction in pathogenic bacteria in the ulcers. This slow-release biopolymer was safe and of possible benefit in the management of refractory wounds, and the apparent utility of bacteriophages was supported in this setting (Markoishvili et al., 2002).
	Prospective, randomized, double-blind, controlled phase I study for the safety and efficacy of treatment of venous leg ulcers was conducted by the Southwest Regional Wound Care Centre in Lubbock, Texas, USA (in 2006–2008).	Once a week for 12 weeks topical application of a cocktail of 8 lytic bacteriophages against *P. aeruginosa, S. aureus*, and *E. coli*. (named WPP-201), developed by Intralytix Inc., USA.	No safety concerns regarding bacteriophage treatment (Rhoads et al., 2009).
*P. aeruginosa*	Twenty-four patients suffering from otitis media caused by antibiotic refractory *P. aeruginosa*. A double-blind, placebo-controlled initial phase I/II clinical trial targeting chronic external ear infections (in 2006–2007).	Application of bacteriophage mixture (Biocontrol Ltd., UK)	The results of the treatment of half of them treated with a single dose of bacteriophage mixture confirmed that phage administration was safe. A significant reduction of clinical symptoms at day 42 in the bacteriophage treated group was observed (55% of total clinical score at day zero) compared to the control group (104%). It was accompanied by a 76% decrease in mean count of bacteria in samples taken from the patients' ears 6 weeks after phage application, whereas in controls a 9% increase was observed (Wright et al., 2009).
*E. coli* or *P. aeruginosa*	*E. coli* or *P. aeruginosa* burn wound infections. A phase I/II randomized multi-center clinical trial involving 11 different burn units located in France, Belgium and Switzerland. This four-arm study involves 220 patients.	Two topically applied therapeutic phage cocktails (PP0121 and PP1131)	Its primary endpoint is the time for reduction of the targeted bacterial load in wound burns with a specifically designed microbiological procedure (Gabard et al., 2015).

## Phages active against *Propionibacterium acnes*

Bacteriophages are a viral component of the skin microbiome, but the knowledge about them in this environment is scant (Hannigan and Grice, 2013), probably because of the colonization of a specific ecological niche (anaerobic microenvironment). Since 1964 it has been known that bacteriophages are one of the components of the human skin community (Brzin, 1964). Webster and Cummins reported in 1978 that 18% of *P. acnes* isolates carried bacteriophages. In the skin microenvironment where there is a lack of bacteria other than *P. acnes* and their phages, *P. acnes* phages are proved to be closely related. The characteristics of described *P. acnes* bacteriophages are presented in Table 2.

**Table 2:** **Described phages that are active against *Propionibacterium acnes***.

**Phage symbol, total number of isolated phage strains**	**Classification in family**	**Brief characteristics**	**Host range and specificity of action**	**Possible use in phage therapy**
PA6	*Siphoviridae*	Lytic (lack of lysogeny genes). Phage isolated from skin scrub wash sample from patient. Phage produces clear plaques with turbid centers.	Able to lyse *P. acnes* strains, but not able to lyse other strains that are a part of the skin microbiome: *Propionibacterium granulosum, Propionibacterium avidum, Staphylococcus epidermidis*, Corynebacterium bovis (Farrar et al., 2007)	High specificity only against *P. acnes* and lytic life cycle may predispose to use of this phage in the therapy of acne.
PAC1-PAC10	Not done	Pseudolysogenic life cycle.	Lysis of *P. acnes* strains, but not lysis of *P. acidipropionici, P. avidum, P. cyclohexanicum, P. jenseni*i, *P. thoenii, P. freudenreichii. Narrow lytic spectrum*.	In Cetomacrogol cream aqueous concentration of phage for potential application in topical treatment of acne (Brown et al., 2016).
		Phage isolated from bacteria from skin swab sample from patient. It did not contain bacterial virulence factors.		
PAD2-PAD48, PAS2-PAS52	*Siphoviridae*	Presence of pseudolysogeny. Do not confer superinfection immunity.	Species specific. Only infect *P. acnes*, not other strains closely related to *Propionibacterium* (Lood et al., 2008).	Probably bad candidate for phage therapy.
P1.1, P9.1, P14.4, P100A, P100D, P100.1, P101.A, P104.A, P105	*Siphoviridae*	Probably the presence of pseudolysogeny (lack of lysogeny-related genes) phages. Isolated from healthy subjects and patients with acne. Lack of genetic diversity.	Broad range of clinical isolates, phage immunity if present is connected with the presence of chromosomally encoded elements.	Marinelli et al. (2012) suggested use of endolysin—peptidoglycan hydrolases that are bacteriophage-encoded antimicrobial peptides to treat bacterial infection.
48 phages, e.g., PHL111M01, PHL071N05, PHL060L00, PHL073M02	*Siphoviridae*	Pseudolysogenic and/or life cycle. 21 phages were isolated from patients with acne, 27 from healthy volunteers. Phages have limited diversity in genome.	*P. acnes* strains (including clades IA-1, IA-2, IB-1, and IB2) were susceptible to all 15 tested phages. But strains of clade IB-3, II, II were highly resistant to phages. Moreover, *P. granulosum* and *P. avidum* were resistant to all tested phages. Two strains of *P. humerusii* were susceptible to all tested phages, one was susceptible to 10 from 15 phages. Activity of *P. acnes* phages includes bacterial strains that are closely related to *Propionibacterium* species.	The authors suggested that the isolated phages may be used in modulation of *Propionibacterium* populations in human skin (Liu et al., 2015).
9 phages: from P-a-1 to P-a-9	Not done	Lytic phages, lysogenic ones were not detected. P-a1 to P-a7 were isolated from plaques on *P. acnes* lawn, but P-a-8 and p-a-9 came from sewage.	Both Gram-positive and Gram-negative strains from genera other than *Propionibacterium* were not lysed by these bacteriophages (Zierdt, 1974).	Bacteriophages were used to distinguish *C. ances* from *C. avidium* and *C. granulosum*. Lytic life cycle may predispose to use of this phage in the therapy of acne.
15 phages	Polyhedral heads with flexible unsheathed tails	Lysogenic, induced with mitomycin C from 17% *of P. acnes* strains	Isolated from *P. acnes* of healthy individuals. Strains of *P. acnes* belonging to serotype I were more susceptible to phage than those from serotype II (Webster and Cummins, 1978).	Probably bad candidate for phage therapy because of lysogenic cycle.
12 phages	Not done	Lysogenic phages. Phages isolated from skin swab sample from patient.	Phage of varying host range, but none which lyses all subtypes of *P. acnes*. (Neely et al., 2008).	Bad candidate for phage therapy because of lysogenic cycle.

Antibiotic resistance of *P. acnes* is emerging (Liu et al., 2014). Liu et al. (2014) isolated a bacterial strain from facial acne (nose skin) of a patient who had not previously applied an antibiotic for acne. It was observed that the isolated HL411PA1 strain was resistant to most antibiotics (including tetracycline, clindamycin, and erythromycin), which may confirm the presence of antibiotic resistance.

Liu et al. (2015) hypothesized that bacteriophages may play an important role in health and disease, because they are the main component of the human microbiota, and therefore they can modulate the bacterial community, especially in the case of human skin. In a pilosebaceous unit of skin it was found that the ratio between phages and bacteria is approximately 1:120, but it may vary (Fitz-Gibbon et al., 2013). Liu et al. (2015) analyzed 48 *P. acnes* phage metagenomes, and, based on this analysis, they found that human skin is colonized most commonly by one strain (Liu et al., 2015). It was also demonstrated that the transmission of phages between individuals is possible. Interestingly, phages active against *P. acnes* were isolated more frequently from skin of healthy volunteers than from patients suffering from acne, which may indicate that the role of phages in human skin is regulatory. One phage strain may dominate in the *P. acnes* phage population. It was also observed that some groups of tested phages were identified in different people, which may indicate that there is a pool of phages that may be shared in the human population. Furthermore, the transmission of skin phages is likely between closely related people. Based on their observations, the authors suggested that in designing phage therapy the composition of individual microbiome structure should be taken into consideration.

Marinelli et al. (2012) isolated 11 *P. acnes* phages from sebaceous follicles of healthy skin. The isolated phages were highly homogeneous and showed no genetic diversity, which the authors linked to their unique habitat. The tested phages had features (lytic lifecycle, lack of lysogeny-related genes, and the presence of endolysin-encoding genes) that make them an ideal tool for phage therapy of acne.

## *P. acnes* phages' ability to kill the host strain

The application of bacteriophages in the therapy of acne shows initial promise *in vitro* (Neely et al., 2008; Brown et al., 2016). *P. acnes* phages from skin of patients suffering from acne have been isolated by Brown et al. (2016). Phages active against *P. acnes* were found in both the gastrointestinal tract and the oral cavity (Willner et al., 2011; Sharon et al., 2013). The authors applied in a semi-solid preparation—Cetomacrogol cream, with concentration of 2.5 × 10^8^ pfu/ml per gram—phages that remained active for 90 days when the preparation was stored in a light-protected place at 4°C (Brown et al., 2016). The non-ionic cream-based preparation was chosen because it excludes potential occurrence of interactions of ions with phage particles. These properties, especially stability and its easy-to-use form, ensure this preparation's potential for topical treatment of skin *P. acnes* infections. Benefits were observed from using the cream form, which can reduce the impact of harmful side-effects that are observed in common therapy of acne. Moreover, the use of Cetomacrogol moisturizing cream may allow the contact of phage particles—which are pharmaceutically active components—with skin areas infected with *P. acnes*, which may improve the effectiveness of the suggested therapy. In this study it was found that the cocktail of phages did not result in higher inhibition of bacteria when compared to application of a single phage, but due to the potential reduction of bacterial resistance, its use was recommended.

It was demonstrated that phages may be formulated in a cream (O'Flaherty et al., 2005), as well as in a water-oil nanoemulsion (Esteban et al., 2014). The latter formulation—bacteriophage containing a nanoemulsion—caused bacteria eradication *in vivo* 10 days after preparation. Markoishvili et al. (2002) reported application of PhagoBioDerm, which contains ciprofloxacin, α-chymotrypsin benzocaine, and bacteriophage based on a biodegradable poly(ester amide)s matrix in patients with wounds/ulcers. Phage application was also demonstrated in humans using an antiseptic gel and paraffin-oil-based lotion to destroy *Acinetobacter baumannii* (Chen et al., 2013).

The treatment of acne with phages in different types of formulations, e.g., creams or liquids, may be important in reducing the overuse of antibiotics, which is the primary means of acne treatment.

## Application of enzymes encoded in phage genome and phage modification

Marinelli et al. (2012) found in phage genomes regions that encode phage endolysin, which is conserved in all tested *P. acnes* phages. These enzymes probably bind to essential elements of the *P. acnes* cell wall and may kill a broad range of *P. acnes* strains. They are essential proteins for the release of progeny, causing lysis of the host bacteria. The authors suggested that it may be possible to develop topical treatment of acne by the use of *P. acnes*-encoded endolysins. They are enzymes encoded in the bacteriophage genome with the ability to cause lysis of the bacterial cell wall peptidoglycan during the phage lytic cycle (Tišáková and Godány, 2014). The enzymes may create opportunities for the construction of genetically engineered enzymes for bacteria elimination, as well as experimental therapies. The detected endolysins in all the characterized *P. acnes* phages were found to be over 95% conserved at the amino acid level (Marinelli et al., 2012). This feature creates the possibility to use any endolysin successfully, destroying every *P. acnes* strain. Moreover, resistance to endolysins has not been observed so far (Nelson et al., 2012). The use of phage lysins as treatment against bacterial infections was demonstrated both *in vitro* and *in vivo*. The PlyG endolysin isolated from the γ phage was applied against *B. anthracis* by Schuch et al. (2002). Susceptibility of *B. anthracis* strains to the purified PlyG lysin indicated that this agent has a narrow bacteriolytic spectrum—especially with high activity against only *B. anthracis* strains. In bacterial culture, application of lysin caused morphological changes of bacterial cells and led to cell lysis. BALB/c mice were intraperitoneally infected with streptomycin-resistant *B. cereus* RSVF1 (with the same proven lytic activity of lysin as in the case of *B. anthracis* strains) and 15 min later were treated with 50 and 150 U of PlyG. The application of lysin significantly rescued mice in comparison to untreated animals. Bacterial resistance to PlyG was not observed *in vitro*, and the authors demonstrated that the RSVF1 strain that became resistant to the phage remained sensitive to PlyG.

Recently, we have suggested that phages armed with homing peptides should allow for their better tissue penetration and achieving *in situ* concentrations necessary for successful eradication of infection and control of inflammation (Górski et al., 2015). This methodology may also be relevant for phage treatment of infections associated with *P. acnes*.

Another approach suggests that phages may be used to improve antibiotic efficacy; in particular, phages may be a tool used for sensitizing antibiotic-resistant bacteria by introducing sensitivity genes (e.g., rpsL ayrA) to bacteria, which may restore drug efficacy (Wikoff et al., 2000; Salmond and Fineran, 2015). The authors believe that antibiotics conjugated with phages may enable the delivery of the drug to target cells and increase the concentration of the drug at the site of infection (Salmond and Fineran, 2015). The issue of a combined treatment with phages and antibiotics has recently been addressed in some detail by Torres-Barceló and Hochberg (2016). This approach has been recommended by some authors (e.g., Chanishvili, 2016). However, we need more data to establish the real value of such therapy, and one should also note that the emergence of double-resistant phage mutants poses a potential threat (Torres-Barceló and Hochberg, 2016).

## Limitations of phage use for elimination of *P. acnes*

Bacteria have an adaptive immune system which is based on a region of DNA called Clustered Regularly Interspaced Short Palindromic Repeats (CRISPR) and CRISPR-associated (*cas*) genes separated by short sequences (Rath et al., 2015; Maxwell, 2016). This system protects bacteria against viruses and other mobile genetic elements. It may be responsible for phage resistance in bacteria. Also *P. acnes* proved to have CRISPR elements, and its role in the phage resistance results from the correlation between these elements and spacer matches with the phage genomes, as suggested by Marinelli et al. (2012). This system, which has also been found in *P. acnes*, may cause phage ineffectiveness (Brüggemann and Lood, 2013).

Another limitation associated with the application of *P. acnes* phages is the high homogeneity within *P. acnes* phages, which may be beneficial with the use of phage formulations of broad activity and application of available ready to use phages of limited activity, avoiding phage typing. However, acquisition of resistance to one phage may result in resistance to others (Brüggemann and Lood, 2013). The effect of *P. acnes* phages' homogeneity may limit the efficacy of the phage therapy.

The term “pseudolysogeny” for *P. acnes* phages was first mentioned in 2011 by Lood and Collin (2011). Probably most, if not all, *P. acnes* phages characterized to date display pseudolysogeny (Farrar et al., 2007; Lood et al., 2008; Lood, 2011; Marinelli et al., 2012) and form turbid plaques. Pseudolysogeny is an unstable state in which the phage genome is not integrated into the bacterial genome (Lood and Collin, 2011). Without cell lysis, the phage genome exists in the host cell as an episome, with superinfection immunity of bacteria from phage infection, and phage DNA exists as a subpopulation. Both the lysogenic and pseudolysogenic cycle do not cause direct lysis of bacteria, or may lead to phage resistance, and these phenomena are not beneficial from the therapeutic point of view. Phages specifically active against *P. acnes* strains may shape the skin microbiome composition and influence the health-disease balance. Moreover, they do not integrate into the host chromosome and transfer bacterial pathogenicity genes, which is an advantage in their use as therapeutics.

There are no data in the available literature regarding phage application against skin infections caused by *P. acnes* in an animal model, while the results obtained *in vitro* do not necessarily translate into the situation *in vivo*. This situation does not facilitate prompt clinical application of phages. On the other hand, phages intended for topical use in the treatment of acne could be used as a medical application which could make the process of their registration more straightforward.

The potential of phage therapy in the treatment of acne has been highlighted by a recent article that emphasized the need for more prudent use of antibiotics in this condition, as well as an urgent need to search for alternative treatments. The authors also draw attention to the changing concept of acne, where inflammation appears to play a prominent role in its pathology. This constitutes another strong argument for the potential application of phages in acne, as the anti-inflammatory action of phage therapy is well documented (Górski et al., 2012).

## Concluding remarks

Phage therapy offers a real chance for patients suffering from chronic infections caused by antibiotic-resistant bacteria. This therapy may replace or supplement conventional antibiotic therapy, help eliminate antibiotic resistance of bacterial strains, and eliminate deleterious effects of chemical antibiotics.

There is a need to develop phage therapy of acne, but more research is needed to understand bacteria-bacteriophage interactions in the skin community to obtain comprehensive knowledge on how to use phages to combat *P. acnes* when it becomes pathogenic (in the pathogenesis of acne vulgaris). Phages should gain acceptance and be widespread as an antibiotic supplement or an alternative in the treatment of infections caused by antibiotic-resistant bacteria, including those whose etiological agent is *P. acnes*. There are also some limitations of the potential application of anti-*P. acnes* phages. Those include their high homogeneity which may cause difficulties in identifying other phages if phage resistance develops. In addition, the lack of data on phage application in skin infections caused by *P. acnes* may delay such clinical application.

## Author contributions

EJ and BW drafted the main part of the manuscript. MŻ prepared a part of the manuscript and Table 2. RM, SL, and MŁS prepared parts of the manuscript. AG gave support and conceptual advice at all stages of manuscript preparation. All authors revised the manuscript.

## Funding

This work was financially supported by the project “Innovative Bacteriophage Preparation for the Treatment of Diabetic Foot” no. POIG.01.03.01-02-048/12 funded by The National Centre for Research and Development. This work was also supported by Wrocław Center for Biotechnology under the Program the Leading National Research Center (KNOW) for the years 2014–2018 granted by the Minister of Science and Higher Education.

### Conflict of interest statement

The authors declare that the research was conducted in the absence of any commercial or financial relationships that could be construed as a potential conflict of interest.
